# Mitochondrial COI and morphological specificity of the mealy aphids (*Hyalopterus* ssp.) collected from different hosts in Europe (Hemiptera, Aphididae)

**DOI:** 10.3897/zookeys.319.4251

**Published:** 2013-07-30

**Authors:** Rimantas Rakauskas, Jekaterina Havelka, Audrius Zaremba

**Affiliations:** 1 Department of Zoology, Vilnius University, M. K. Čiurlionio 21/27, LT-03101, Vilnius, Lithuania

**Keywords:** Europe, *Hyalopterus amygdali*, *Hyalopterus pruni*, *Hyalopterus persikonus*, molecular phylogeny, mitochondrial COI, morphological key to species

## Abstract

Forty three European population samples of mealy aphids from various winter and summer host plants were attributed to respective species of *Hyalopterus* by means of their partial sequences of mitochondrial COI gene. Used *Hyalopterus* samples emerged as monophyletic relative to outgroup and formed three major clades representing three host specific mealy aphid species in the Neighbor joining, Maximum parsimony, Maximum likelihood and Bayesian inference trees. *Hyalopterus pruni* and *Hyalopterus persikonus* emerged as a sister species, whilst *Hyalopterus amygdali* was located basally. Samples representing different clades in the molecular trees were used for canonical discrimination analysis based on twenty two morphological characters. Length of the median dorsal head hair enabled a 97.3 % separation of *Hyalopterus amygdali* from the remaining two species. No single character enabled satisfactory discrimination between apterous viviparous females of *Hyalopterus pruni* and *Hyalopterus persikonus*. A modified key for the morphological identification of *Hyalopterus* species is suggested and their taxonomic status discussed.

## Introduction

Mealy aphids of the genus Hyalopterus Koch are reported to be serious pests of stone fruits all over the World (Barbagallo et al. 1997, Blackman and Eastop 2000, Lozier et al. 2009). Therefore, their morphology, biology, systematics, evolution, invasion history and potential harmfulness have been substantially studied (Smolarz 1970, Tscharntke 1989, Mosco et al. 1997, Poulios et al. 2007, Lozier et al. 2008, Tewksbury et al. 2002, Penvern et al. 2010, Symmes et al. 2012; for more and earlier references see Blackman and Eastop 2000). Nonetheless, the species level classification of mealy aphids remains unclear despite the long lasting debate. Since the very beginning, mealy aphids inhabiting various prunoideous plants have been described as a single species, *Hyalopterus pruni* (Geoffroy, 1762). Later on, almond inhabiting aphids were separated as *Hyalopterus amygdali* Blanchard, 1840. Such a viewpoint has been subjected for a long lasting controversy (e.g. Börner 1952, Shaposhnikov 1972, Eastop and Hille Ris Lambers 1976, Stroyan 1984, Heie 1986, Remaudiere and Remaudiere 1997). Recently, in addition to the two above mentioned species, *Hyalopterus persikonus* Miller, Lozier and Foottit, 2008 has been separated from *Hyalopterus amygdali* by Lozier et al. (2008). For the present, three host plant associated Hyalopterus species are recognized. All three might inhabit reeds (Phragmites) as a summer hosts, but are different in their winter host specificity: *Hyalopterus amygdali* is associated with almonds, whilst *Hyalopterus pruni* and *Hyalopterus persikonus* with plums and peaches, respectively. Nonetheless, apricot has been reported as a shared resource among the three *Hyalopterus* species supporting the possibility of interspecific hybridization (Lozier et al. 2007, Poulios et al. 2007, Lozier et al. 2008). *Hyalopterus* species, although well-defined on molecular level (Lozier et al. 2008), still remain difficult to separate by their morphological characters (Basky and Szalay-Marszό 1987, Blackman and Eastop 1994, 2000, 2006), including the most recent identification key (Lozier et al. 2008). For example, mealy aphids, collected on apricots in Lithuania, run to *Hyalopterus amygdali* in the key of Blackman and Eastop (2000), but appeared difficult to identify by means of the key suggested by Lozier et al. (2008) (Kudirkaitė-Akulienė and Rakauskas 2009). Moreover, the above keys do not concern mealy aphid populations on summer hosts, reeds. Host plant mediated developmental pathways might influence morphological characters, therefore, samples from reeds must be included in the analysis, together with those from stone fruit crops.

The aim of this study was to elaborate morphological identification key of the genus *Hyalopterus* based on the material from Europe that was identified by means of partial CO-I sequences.

## Material and methods

### Material studied

Forty three population samples of mealy aphids from five European countries were collected from various winter and summer host plants (Table 1). The entire data set has been subdivided: 21 samples (bolded in Table 1) were used for canonical discrimination procedures and subsequent evaluation of the received discrimination functions was performed on remaining 22 samples.

**Table 1.** Aphid material used in the present study. Samples used for the morphological discrimination analysis with *a priori* specified group membership are given in bold.

**Place, date, collection No**	**GenBank Accession No**
***Prunus domestica*** **(plum)**	
**Galata, Bulgaria, 2012.06.18, z12-101**	JX943533
**Costinesti, Romania, 2012.06.13, z12-67**	JX943536
**Gilau, Romania, 2012.06.19, z12-114**	JX943537
**Toplita, Romania, 2012.06.10, z12-46b**	JX943538
**Constanta, Romania, 2012.06.14, z12-78**	JX943539
**Valu lui Traian, Romania, 2012.06.14, z12-77**	JX943540
**Michalovce, Slovakia, 2012.06.08, z12-43a**	JX943545
**Mezopeterd, Hungary, 2012.06.20, z12-121**	JX943541
**Derecske, Hungary, 2012.06.20, z12-123**	JX943542
**Gemzse, Hungary, 2012.06.08, z12-44**	JX943543
Jieznas, Prienai distr., Lithuania, 2012.05.30, 12-24	JX943544
Daugai, Alytus distr., Lithuania, 2012.05.30, 12-31	JX943547
Ignalina, Ignalina distr., Lithuania, 2012.06.19, 12-65	JX943549
***Prunus cerasifera*** **(cherry plum)**	
Ditrau, Romania, 2012.06.11, z12-52	JX943534
Gheorheni, Romania, 2012.06.11, z12-53	JX943535
Blagojevgrad, Bulgaria, 2012.06.25, 12-81	JX943550
Alytus, Alytus distr., Lithuania, 2012.05.30, 12-28	JX943546
Eišiškės, Šalčininkai distr., Lithuania, 2012.06.13, 12-41	JX943548
***Prunus cerasifera*** **var.** ***Pissardii*** **(red plum)**	
Costinesti, Romania, 2012.06.13, z12-65	JX943553
***Prunus armeniaca*** **(apricot)**	
Costinesti, Romania, 2012.06.15, z12-88	JX943551
Murfatlar, Romania, 2012.06.13, z12-64	JX943531
Vama Veche, Romania, 2012.06.16, z12-93	JX943552
Mezopeterd, Hungary, 2012.06.20, z12-120	JX943555
Kairėnai, Vilnius distr., Lithuania, 2010.07.01, z10-5	JX943558
***Prunus persica*** **(peach)**	
**Goron, Bulgaria, 2012.06.09, z12-111**	JX943519
Bucuresti, Romania, 2012.06.13, z12-58	JX943521
**Constanta, Romania, 2012.06.14, z12-79**	JX943522
**Costinesti, Romania, 2012.06.15, z12-86**	JX943523
**Murfatlar, Romania, 2012.06.13, z12-63**	JX943524
**Pieta Porta Alba, Romania, 2012.06.14, z12-70**	JX943525
**Valu lui Traian. Romania, 2012.06.14, z12-75**	JX943526
Mezopeterd, Hungary, 2012.06.20, z12-119	JX943527
**Szikso, Hungary, 2012.06.20, z12-124**	JX943528
**Csobad, Hungary, 2012.06.20, z12-126**	JX943529
**Foro, Hungary, 2012.06.20, z12-127**	JX943530
***Prunus persica*** **var.** ***nectarina*** **(nectarine)**	
Pieta Porta Alba, Romania, 2012.06.14, z12-73	JX943520
***Prunus dulcis*** **(almond)**	
**Varna, Bulgaria, 2012.06.18, z12-104**	JX943517
**Varna, Bulgaria, 2012.06.18, z12-108**	JX943518
***Prunus maritima*** **(beach plum)**	
Kairėnai, Vilnius distr., Lithuania, 2010.07.01, z10-4	JX943557
***Phragmites australis*** **(common reed)**	
Vama Veche, Romania, 2012.06.16, z12-91	JX943532
Biharkeresztes, Hungary, 2012.06.20, z12-118	JX943554
Baltupiai, Vilnius, Lithuania, 2010.06.30, z10-1	JX943556
Palanga, Klaipėda distr., Lithuania, 2010.07.15, z10-24	JX943559

### DNA extraction, PCR amplification and sequencing

For molecular analysis, a single aphid individual from one sampled plant was considered as a unique sample. Total genomic DNA was extracted from a single aphid using the DNeasy Blood & Tissue kit (Qiagen), which involved at least a 2 h digestion of tissue with proteinase K. Partial sequences of mitochondrial COIwere PCR-amplified using previously published primers (Turčinavičienė et al. 2006). PCR amplification was carried out in a thermal cycler (Eppendorf) in 50 µl volumes containing 1–2 µl genomic DNA, 5 µl of each primer (10 µM), 5 µl of PCR-reaction buffer, 5 µl of dNTP mix (2mM each), 4–8 µl of 25mM MgCl_2_ and 1.25 U of AmpliTaq Gold 360 polymerase (5U/µl) and ddH_2_O to 50 µl. The cycling parameters were as follows: denaturizing at 95°C for 10 min (1 cycle), denaturizing at 95°C for 30”, annealing at 49°C for 30” and extension at 72°C for 30” (32–37 cycles in total), and a final extension for 5 min (1 cycle). PCR products were subjected to electrophoresis on 2% TopVision agarose (Fermentas, Lithuania), stained with ethidium bromide and sized against a MassRuler Low Range DNA ladder (Fermentas, Lithuania) under UV light. PCR products were purified and sequenced at Macrogen Europe (Amsterdam, the Netherlands). The amplification primers were also used as sequencing primers. DNA sequences for each specimen were confirmed with both sense and anti-sense strands and aligned in the BioEdit Sequence Alignment Editor (Hall 1999). Partial sequences of COI gene were tested for stop codons and none were found. The sequence data have been submitted to the GenBank, Accession numbers JX943517-JX943559.

### Analysis of DNA sequences

Forty three sequences of three *Hyalopterus* species were analyzed. Sequences of *Aphis gossypii* Glover, 1877 (Aphidini) and *Nasonovia ribisnigri* (Mosley, 1841) (Macrosiphini) were selected as outgroups for the phylogenetic analyses, which included Neighbor joining (NJ), Maximum parsimony (MP), Maximum likelihood (ML) and Bayesian inference in phylogeny (BI). NJ, MP and ML analyses were performed using MEGA 5 (Tamura et al. 2011). For NJ analysis Kimura 2-parameter (K2P) model of base substitution was used. Bootstrap values for NJ, MP and ML trees were generated from 1000 replicates. For ML analysis Tamura 3-parameter model with Gamma distribution (T92+G) was selected by MEGA 5 model selection option (Tamura et al. 2011). Bayesian analysis was conducted in MrBayes 3.2.1 (Ronquist and Huelsenbeck 2003) using General Time Reversible model with Gamma distribution (GTR+G), which was selected by jModeltest (Posada 2008). Four simultaneous chains, 3 heated and 1 “cold”, were run for 3 000 000 generations with tree sampling every 1000 generations. The topologies obtained by NJ, MP, ML and BI were similar, so only ML tree is shown with values of NJ/MP and ML/BI bootstrap support and posterior probabilities indicated above and below branches respectively.

### Morphological study and discrimination analysis

Samples representing different clades in the molecular trees were used for canonical discrimination analysis: 2 samples from almond (*Hyalopterus amygdali* clade), 10 samples from cultivated plums (*Hyalopterus pruni* clade), and 9 samples from peaches (*Hyalopterus persikonus* clade) (Table 1).

Based on the earlier references (Poulios et al. 2007, Lozier et al. 2008), twenty two metric (in mm) characters were studied:

A2L – length of antennal segment 2; A2W – width of antennal segment 2; A3BW – basal width of antennal segment 3; A3L – length of antennal segment 3; A3SL – length of the longest hair on antennal segment 3; A4L – length of antennal segment 4; A5L – length of antennal segment 5; A6BL – length of basal part of antennal segment 6; A6TPL – length of terminal process of antennal segment 6; AT8SL – length of submedian hair on abdominal tergite 8; BL – body length (excluding cauda); CL – length of cauda; DT3L – length of the second segment of hind tarsus; F3L – length of hind femur; FSL – length of the frons hair; HW – width of the head across eyes; MDHSL – length of median dorsal head hair; MDHSW – distance between the bases of median dorsal head hairs. SL – length of siphunculus; T3L – length of hind tibia; URL – length of ultimate rostral segment; URW – basal width of ultimate rostral segment.

Measurements of the slide-mounted apterous viviparous females were performed by means of interactive measurement system Micro-Image (Olympus Optical Co. GmbH). STATISTICA 8 version software (Statsoft 2007) was exploited for data analysis. Pearson’s correlation coefficients were calculated to evaluate the correlation of morphometric characters with body length. Characters with strong (| r | ≥ 0.50) statistically significant (p<0.05) correlation with body length were removed from the further analysis: BL (r=1.00), F3L (r=0.58), T3L (r=0.59), A2L (r=0.57), HW (r=0.51). Remaining seventeen characters were used for forward stepwise discriminant analysis with host plant species as grouping variable followed by canonical analysis. Discriminant analysis was conducted in three steps. The first step was performed to discriminate between the all three mealy aphid species emerged in the COI dendrogram (*Hyalopterus amygdali*, *Hyalopterus persikonus* and *Hyalopterus pruni*). The second step was carried out to discriminate between *Hyalopterus persikonus* and non- *Hyalopterus persikonus* (*Hyalopterus amygdali* and *Hyalopterus pruni*) samples. The third step of the discriminant analysis was performed on *Hyalopterus amygdali* - *Hyalopterus pruni* data set (*Hyalopterus persikonus* samples excluded) to separate almond and plum mealy aphid species. Canonical scores were visualized as scatter plots. The morphological interrelationships among different samples were examined using hierarchical cluster analysis based on squared Mahalanobis distances (linkage method – UPGA).

Characters that contributed most in canonical discrimination functions were evaluated as having potential for species separation. The eventual species identification key based on these morphological characters and host plant information was constructed. Afterwards, it was applied on mealy aphid samples that were not used for the construction of the identification key (Table 1).

## Results

### Partial sequences of mitochondrial (COI)

Lozier et al. (2008) reported partial COI sequences being the most variable in *Hyalopterus* aphids and suggested them as a possible tool for the identification of the mealy aphid species complex. Forty three partial COI sequences of 3 *Hyalopterus* species from 5 countries were included in analysis. The alignment contained 564 bases in final set with 79 variable sites, 35 of which appeared parsimony informative. The sequences were heavily biased towards A and T nucleotides. The average base composition was A = 34.3 %, C = 14.1 %, G = 12.0 % and T = 39.7 %. The overall transition/transversion ratio R = 2.805 for all sites.

**Figure 1.**
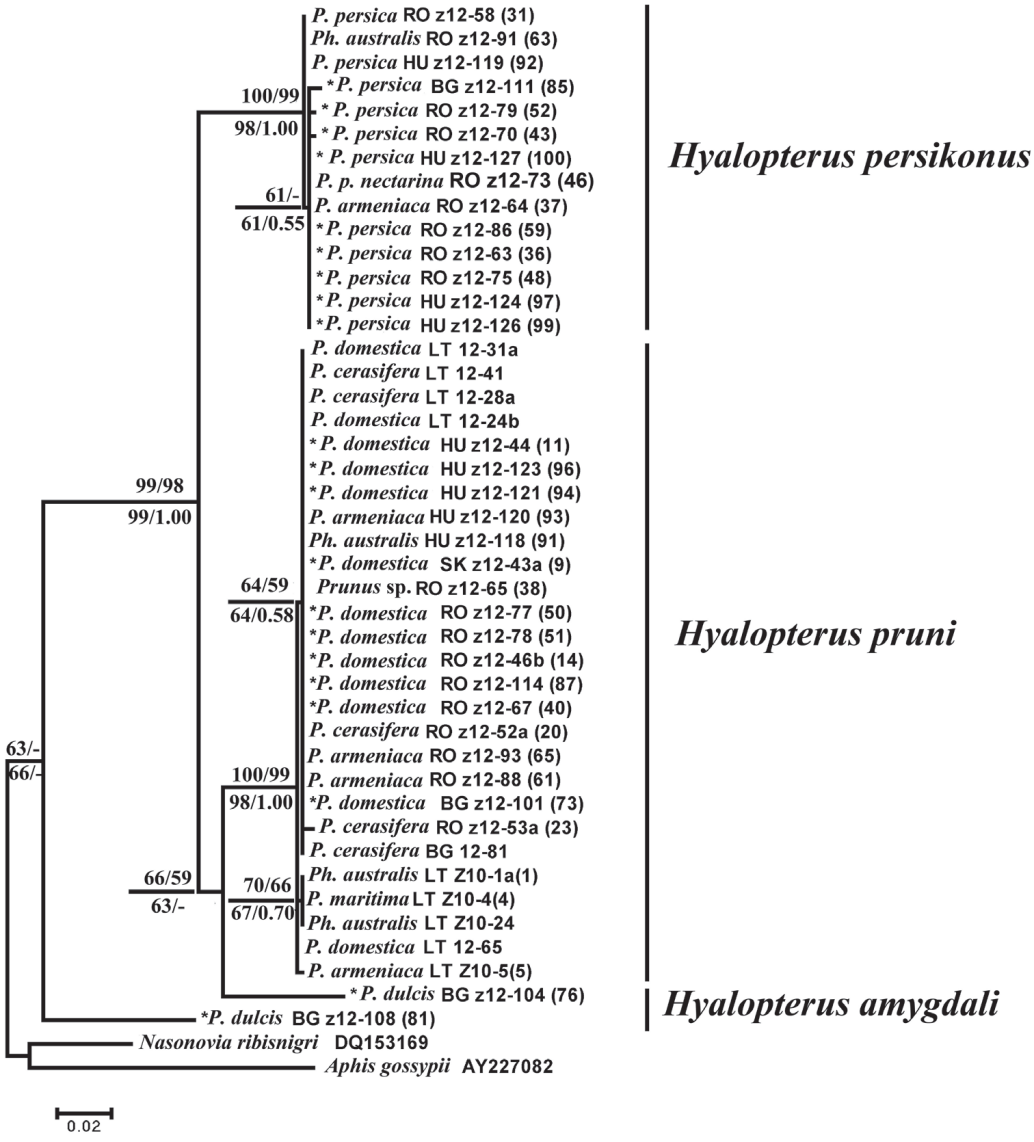
Maximum likelihood (ML) tree showing phylogenetic relationships among three *Hyalopterus* species based on partial sequences of mitochondrial COI (564 positions in final set). Numbers above branches indicate support of NJ (left) and MP (right) bootstrap test with 1000 replicates, and numbers below branches indicate support of ML (left) bootstrap test with 1000 replicates and posterior probabilities of BI analysis (right). Samples used for the discriminant analysis with *a priori* specified group membership followed by the construction of identification key are asterisked (*). The remaining samples were used for the *post hoc* classification. Sample numbers are the same as given in Table 1, together with the abbreviated symbol of respective country: **BG** Bulgaria, **HU** Hungary, **LT** Lithuania, **RO** Romania, **SK** Slovakia.

The maximum parsimony (MP) analysis of partial COI sequences resulted in 425 equally parsimonious trees (length = 152, CI=0.76, RI=0.95). ML tree (T92+G model) showed similar topology, the same as NJ analysis (Kimura 2-parameter distances) and BI (GTR+G model) analyses. NJ, MP and ML bootstrap values over 50 % together with BI posterior probabilities over 0.50 are given at respective nodes of the same tree in Fig. 1. One can ensure that used *Hyalopterus* samples emerge as monophyletic relative to outgroup and form three major clades representing three host specific mealy aphid species. *Hyalopterus pruni* and *Hyalopterus persikonus* are placed as a sister species, whilst *Hyalopterus amygdali* is located basally.

### Morphology

**Figure 2.**
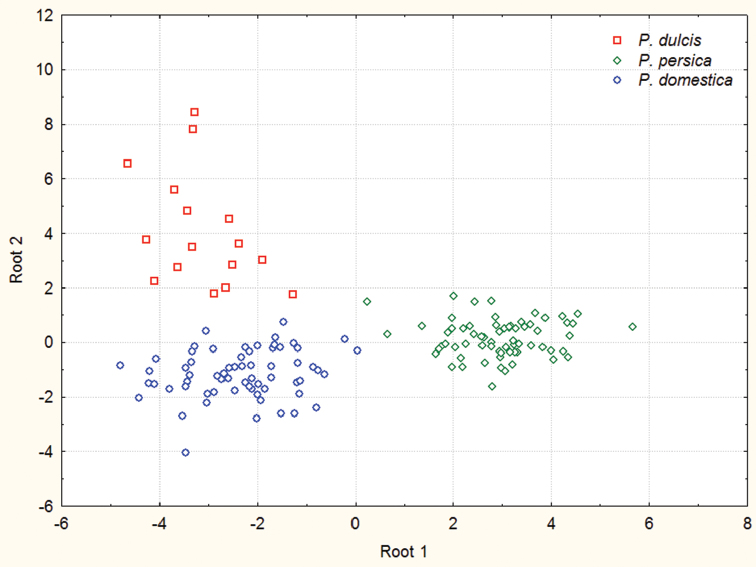
Scatter-plot of the individual canonical scores of the first two canonical variates discriminating 21 samples of *Hyalopterus* collected from different host plants in five European countries (Bulgaria, Hungary, Lithuania, Romania, Slovakia).

The scatter plot of the first two canonical variates for samples from 18 different geographical localities representing three mealy aphid species (apterous viviparous females) is shown in Fig. 2. All individuals were reclassified correctly into their *a priori* specified groups. The following characters proved to be important predictors when separating between three *Hyalopterus* species: MDHSL, URW, T3L/CL (Table 2). The *post hoc* classification of samples gave 96.7 % correct identification of *Hyalopterus persikonus* (n=46), 100 % of *Hyalopterus amygdali* (n=10) and 99% of *Hyalopterus pruni* (n=94) specimens.

**Table 2.** Contribution of eleven morphological characters to the canonical functions discriminating 23 European samples of *Hyalopterus*. Character abbreviations the same as in the text (Material and methods).

	**Wilks’ Lambda**	**Partial Wilks’ Lambda**	**F-remove (2,135)**	**p-level**	**Toler.**	**1-Toler. (R-Sqr.)**
**T3L/CL**	0,05	0,66	34,70	0,00	0,71	0,29
**MDHSL**	0,04	0,81	15,40	0,00	0,14	0,86
**URW**	0,04	0,82	14,33	0,00	0,86	0,14
**URL**	0,04	0,89	8,37	0,00	0,81	0,19
**DT3L**	0,04	0,97	1,98	0,14	0,69	0,31
**A6TPL**	0,04	0,86	11,14	0,00	0,60	0,40
**MDHSW**	0,06	0,58	48,13	0,00	0,12	0,88
**MDHSW/MDHSL**	0,06	0,58	49,50	0,00	0,07	0,93
**A5L**	0,04	0,90	7,57	0,00	0,40	0,61
**SL**	0,04	0,92	6,30	0,00	0,75	0,25
**A6BL**	0,04	0,96	3,04	0,05	0,60	0,40

To discriminate between apterous viviparous females of *Hyalopterus persikonus* and non- *Hyalopterus persikonus* (*Hyalopterus amygdali* and *Hyalopterus pruni*) samples the following canonical function (for character acronyms see above) was obtained: 74.6150*URW-1.2696*T3L/CL+1. The values of canonical scores were >0 for *Hyalopterus persikonus* and <0 for *Hyalopterus amygdali* + *Hyalopterus pruni*. This combination of canonical variables separated 100 % of *Hyalopterus persikonus* (n=71) specimens involved in the analysis with *a priori* specified group membership. The *post hoc* classification gave 94.4 % correct identification of *Hyalopterus persikonus* (n=46) specimens.

To discriminate between apterous viviparous females of *Hyalopterus amygdali* and *Hyalopterus pruni* samples the following canonical function (for character acronyms see above) was obtained: -2.2645*SL-18.6609*MDHSL+1. The values of canonical scores were >0 for *Hyalopterus amygdali* and <0 for *Hyalopterus pruni*. This combination of canonical variables separated 94.5 % of *Hyalopterus amygdali* (n=18) and 100% of *Hyalopterus pruni* (n=67) specimens involved in the analysis with *a priori* specified group membership. The *post hoc* classification gave 100 % correct identification of *Hyalopterus amygdali* (n=10) and 94.7% of *Hyalopterus pruni* (n=94) specimens.

Out of eleven morphological characters included in the canonical function discriminating between sampled apterous viviparous females of mealy aphidspecies complex, the length of median dorsal head hair (MDHSL) enabled separation of 97.3 % *Hyalopterus amygdali* specimens. Namely, the lengths of median dorsal head hair from 0.026 to 0.039 mm were characteristic of *Hyalopterus amygdali*, whilst 0.036 – 0.067 mm – for other two species. Yet we failed to find any single character or ratio enabling satisfactory discrimination between apterous viviparous females of *Hyalopterus pruni* and *Hyalopterus persikonus*. For the present, the following morphological identification key might be suggested to identify apterous viviparous females of the mealy aphid species complex.

### Species key (apterous viviparous females)

**Table:** 

1	Canonical discrimination function 74,6150*URW - 1,2696*T3L/CL + 1 value exceeding 0. Setae on frons stout. On peaches, nectarines, apricots or reeds	*Hyalopterus persikonus*
–	Canonical discrimination function value less than 0. Setae on frons filiform. On almonds, plums, apricots or reed	2
2	Length of the median dorsal head hair (MDHSL) 0.026 – 0.039 (average 0.031) mm. Canonical discrimination function -2.2645*SL - 18.6609* MDHSL + 1value exceeds 0. On almond or reeds	*Hyalopterus amygdali*
–	MDHSL 0.036 – 0.067 (0.05) mm. Canonical discrimination function value less than 0. On plums, apricots or reeds	*Hyalopterus pruni*

## Discussion and conclusions

Our analysis shows the morphological separation of mealy aphid species complex being a really difficult task which is in accordance with the earlier references (Poulios et al. 2007; Lozier et al. 2008). Nonetheless, it appeared that certain morphological characters are effective when applied independently on different data. Namely, the length of median dorsal head hair (MDHSL) has been included in the key of Lozier et al. (2008) to separate *Hyalopterus amygdali* from *Hyalopterus pruni*/*Hyalopterus persikonus*. This character enabled separation between *Hyalopterus amygdali* and *Hyalopterus pruni*/*Hyalopterus persikonus* in our analysis also. Ratio hind tibia length/cauda length (T3L/CL) has been employed in the key of Lozier et al. (2008) to discriminate between *Hyalopterus pruni* and *Hyalopterus persikonus*, although they reported remarkable overlapping of this character values in *Hyalopterus pruni* (4.6–8.3, average 6.1) and *Hyalopterus persikonus* (3.7–7.6, average 5.1). This was also the case in our study: 4.47–6.71 (5.46) for *Hyalopterus pruni*, 3.76–5.36 (4.41) for *Hyalopterus persikonus* and 4.73–6.72 (5.29) for *Hyalopterus amygdali*. In addition to the above mentioned characters, our analysis showed the basal width of the ultimate rostral segment being of certain use when discriminating between the mealy aphid species. Its values were 0.059–0.075 mm (average 0.067) for *Hyalopterus pruni*, 0.064–0.083 (0.073) for *Hyalopterus persikonus* and 0.061–0.071 (0.066) for *Hyalopterus amygdali*.

When performing discriminant analyses, the body length should be eliminated from the data set together with characters that have strong and statistically significant correlation with the body length. In our case, when the entire data set of morphological characters was used for discriminant analysis, samples from reeds appeared the most different (not shown). Contrary, after the body length and correlated characters were removed from analysis, samples from reeds scattered amongst samples from plum and peach.

**Figure 3.**
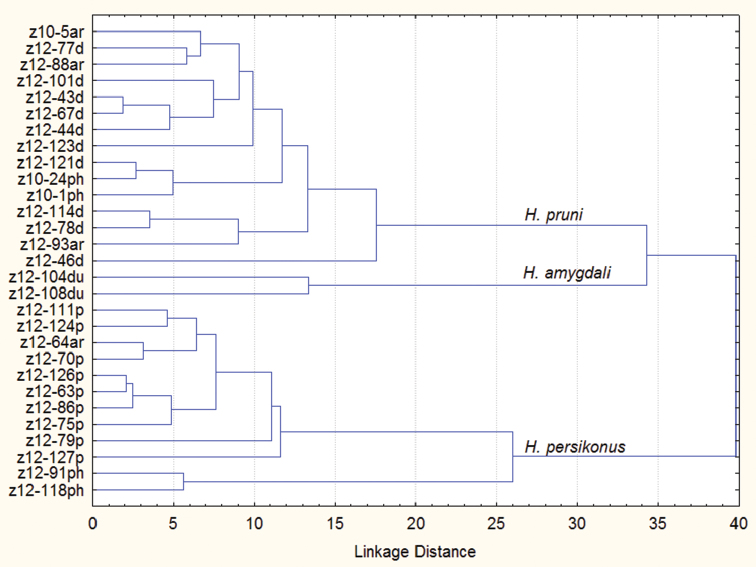
Dendrogram of hierarchical cluster analysis based on 17 morphological characters (squared Mahalanobis distances) using unweighted pair-group average linkage among 29 samples of *Hyalopterus*. Sample numbers the same as in Table 1. **ar** samples from *Prunus armeniaca*, **d** *Prunus domestica*, **du** *Prunus dulcis*, **p** *Prunus persica*, **ph** *Phragmites communis*.

The results of cluster analysis based on morphological data (Figure 3) show *Hyalopterus persikonus* being more distantly related with *Hyalopterus pruni* and *Hyalopterus amygdali*. This contradicts the results of morphological analysis by Poulios et al. (2007) and supports the opinion of Mosco et al. (1997) on the early separation of *Hyalopterus persikonus* from *Hyalopterus pruni/amygdali* stem, which was also supported by the subsequent molecular analyses (Lozier et al. 2007, 2008). Such long lasting controversy might be explained by the fact that all three species share apricot as a winter host (see Lozier et al. (2008) for broader discussion), enabling interspecific gene flow. To clear the matter, precise studies of the host specificity and life cycles of the three taxa (including experimental transfers from plums to reeds and vice versa), together with hybridization trials, are needed. For the present, phylogenetic relationships of the three *Hyalopterus* species remain uncertain.
